# Routine Preoperative Dental Clearance for Total Joint Arthroplasty: Is There a Benefit?

**DOI:** 10.7759/cureus.41352

**Published:** 2023-07-04

**Authors:** Stephanie A Kwan, Vincent Lau, Brian E Fliegel, Colin Baker, Paul M Courtney, Gregory K Deirmengian

**Affiliations:** 1 Orthopaedic Surgery, Jefferson Health New Jersey, Stratford, USA; 2 Orthopaedic Surgery, Rothman Orthopaedic Institute, Philadelphia, USA

**Keywords:** dental clearance, hip and knee arthroplasty, periprosthetic joint infection, infection prevention, adult reconstruction

## Abstract

Background

Periprosthetic joint infections (PJIs) place significant psychological and financial burdens on patients and healthcare systems. One measure to reduce the risk of PJIs is preoperative dental screening, for which there is no current consensus recommendation. This study aims to determine whether there is a difference in the rate of PJI and microorganism profile in patients who did and did not obtain preoperative dental clearance.

Methodology

A retrospective review was conducted among patients undergoing primary total hip arthroplasty and total knee arthroplasty from 2017 to 2021. A cohort of 8,654 patients who underwent routine dental clearance was matched with a cohort of patients who did not. Surgeons who changed their dental clearance protocol were also identified, and the rates of PJIs were compared before and after.

Results

No statistically significant difference was seen in the rate of PJIs between patients who did and did not undergo routine preoperative dental clearance. No statistically significant difference was seen in the rate of PJIs before and after for surgeons who changed their dental clearance protocol. The microorganism profile between the groups was also found to be without differences.

Conclusions

Eliminating dental clearance from routine preoperative clearance does not appear to increase the rates of acute PJIs following elective total joint arthroplasty (TJA) or to change the organism profile of the infections that did occur. It may be reasonable to not require routine preoperative dental clearance or to practice selective dental clearance in patients undergoing elective TJA, especially given the increased financial cost and delay in care experienced by patients.

## Introduction

Periprosthetic joint infections (PJIs) are a serious complication of total joint arthroplasty (TJA). Periprosthetic infections in total hip arthroplasty (THA) patients have a one-year mortality rate of 4.2% and a five-year mortality rate of 21.1% [1]. Total knee arthroplasty (TKA) PJIs have a reported one-year mortality rate of 4.3% and a five-year mortality rate of 21.6% [2]. In addition to their substantial morbidity and mortality, PJIs are associated with a significant economic burden. The estimated annual mean total episode cost per patient of THA PJI and TKA PJI are $88,623 and $116,383, respectively [2,3]. Currently, the number of TJAs each year is projected to rise, and it can be expected that the incidence and economic burden of PJIs will grow accordingly as well [4,5]. Therefore, it is important to mitigate the risk of PJIs by identifying and optimizing modifiable risk factors.

Risk factors for PJIs that have been identified include smoking, poor nutritional status, poor glycemic control, and poor dental health [6]. Dental infections as a potential source for PJI have been controversial in the literature. Some studies show that routine dental procedures and active dental infections cause transient bacteremia, which may then subsequently seed to the prosthesis [7,8]. In some studies, dental procedures were associated with 0-15.9% of PJIs overall and specifically associated with PJIs in 6% of THAs and 11% of TKAs [7-9]. Conversely, other studies have shown no statistically significant relationship between dental pathogens and PJIs [10]. Given the uncertainty in the literature, some orthopedic surgeons require that patients obtain dental clearance before an elective TJA. Data on whether this practice helps mitigate the chance of PJI secondary to a dental pathogen continues to be conflicting [11-14].

When dental clearance is obtained routinely, 18.5% of patients required dental procedures before TJA [11,12]. Pursuing dental clearance before TJA can represent significant financial and psychological burdens for both patients and providers [15,16]. Patients who do not have established dental care may face multiple delays before TJA due to difficulty in scheduling evaluations/procedures and subsequent recovery periods [11,15,16]. Furthermore, when patients finally receive care, they often have out-of-pocket expenses [11,15]. Preoperative invasive dental procedures may also place patients at a higher risk of postoperative complications compared to patients who did not [17,18]. Additionally, no standardized approach to dental clearance has been established creating confusion for providers regarding which procedures are necessary [19]. Surgical staff must allocate valuable time and attention to ensure patient compliance and appropriate communication from the dentists and any failure to do so may result in cancellation of surgery [11,16].

The purpose of our study is to compare the rate of acute PJIs and the microorganism profile of PJIs in primary TJA patients who did and did not obtain preoperative dental clearance. Our secondary goals are to evaluate for any difference in the rates of PJIs in surgeons who changed their dental clearance protocol during the study period. We hypothesize that (1) routine dental clearance does not decrease the rate of infection, (2) routine dental clearance does not affect the prevalence of infections secondary to oral flora, and (3) routine dental clearance does not affect the rates of PJIs in surgeons who changed their dental clearance protocol.

## Materials and methods

Institutional review board approval (#08R.20) from Thomas Jefferson University Hospital was obtained before the start of the investigation. A retrospective review was performed using a large, private academic institution’s database to identify patients who underwent primary THA and TKA from 2017 to 2021 by one of 16 fellowship-trained TJA surgeons. All surgeons were asked about their preoperative dental screening protocols during the timeframe of the study and patient charts were reviewed to confirm these practices. No surgeons in the study used selective dental clearance. A total of three surgeons required preoperative dental clearance in all patients during the timeframe of the study. A total of nine surgeons did not require routine preoperative dental clearance during the timeframe of the study. A total of four surgeons abruptly changed their protocol from requiring preoperative dental clearance to not requiring preoperative dental clearance during the timeframe of the study, and the dates of these changes were confirmed with patient charts.

A retrospective chart review of electronic medical records (EMRs) was performed to collect all relevant patient information, including age at the time of procedure, sex, and medical comorbidities. Patient records were reviewed in both groups to confirm the accuracy of each surgeon’s preoperative protocol. Due to the large number of patients in the study, confirmation of surgeon dental clearance practices was stopped after 10% of the chart reviews showed zero instances of discordance. The cohort was separated into two groups based on preoperative clearance: patients who underwent preoperative dental clearance (DC) and patients who did not undergo preoperative dental clearance (NC). A propensity score was developed using age, sex, body mass index (BMI), and procedure to create an NC patient cohort matched one-to-one to the DC cohort.

EMRs for both groups were then initially screened using International Classification of Diseases 10th Edition (ICD-10) PJI codes T84.50XA, T84.51XA, T84.52XA, T84.53XA, and T84.54XA. A review of all EMRs identified was then performed to confirm the diagnosis of PJI using the 2018 International Consensus Meeting (ICM) criteria [20], the onset of symptoms, microorganisms isolated from cultures, and antibiotics administered. Acute PJI was defined as PJI within 90 days of the primary procedure. We excluded patients with PJI after 90 days of TJA as well as patients with other documented infectious pathology before or within 90 days of TJA.

The data were then compared between the two groups. In addition, any subsequent surgical intervention and/or complication was recorded. The primary outcome measure was the diagnosis of PJI. Secondary outcome measures included the identity of the organism isolated from the culture, whether the organism may have originated from oral flora based on its speciation, and the rate of PJI following the change in dental clearance protocols.

Using the total number of patients and rate of infection of our cohort and assuming an alpha of 0.05 and a power of 0.8, a sample size calculator was used to determine the minimum change in infection rate that could be detected in our study. Following a propensity score match, the data were broken down descriptively based on having a dental clearance or no dental clearance. Continuous data are presented as mean (standard deviation), and categorical data are presented as cell count (%). Normality for all continuous data was assessed via Shapiro-Wilks tests. Mann-Whitney U tests were used to calculate p-values for continuous data, and chi-square tests were used to calculate p-values for categorical data. An analysis was performed following this focusing on the confirmed cases of PJI between the two groups. Rates of PJIs between surgeons were then compared for the DC and NC groups. Lastly, rates of PJIs were compared for surgeons before and after they changed their preoperative protocol. Significance was determined at a p-value <0.05. All statistical analyses were done using R Studio (Version 4.1.2, Vienna, Austria).

## Results

The initial query of the database produced 36,856 patients who underwent primary TJA from 2017 to 2021 at our institution. Preoperative, routine dental clearance was obtained for 8,654 of these patients while the remainder did not undergo clearance. A matched cohort of 8,654 patients total was created. TKA was performed in 64.1% of these patients. Women included 56% of the patients. The mean age at the time of the procedure was 66.4 years. Demographic data are included in Table 1.

**Table 1:** Patient demographics.

Variable	Non-dental clearance	Dental clearance	P-value
	N = 8,654	N = 8,654	
Body mass index	30.4 (5.04)	30.4 (5.07)	0.949
Age	66.4 (9.13)	66.3 (9.41)	0.839
Sex			0.866
Female	4,879 (56.4%)	4,891 (56.5%)	
Male	3,775 (43.6%)	3.763 (43.5%)	
Joint			1.000
Hips	3,107 (35.9%)	3,107 (35.9%)	
Knees	5,547 (64.1%)	5,547 (64.1%)	
Acute periprosthetic joint infection			0.559
No	8,633 (99.8%)	8,628 (99.7%)	
Yes	21 (0.24%)	26 (0.30%)	

A total of 90 patients were identified using the ICD-10 infection codes. Each of these charts was reviewed and 43 of the patients included diagnoses of superficial delayed wound healing (8), drainage (4), hematomas (3), COVID-19 (1), and cases that did not meet the ICM criteria for PJI (27). These patients were not diagnosed with or treated for acute PJIs and were not included in the subgroup of acute PJIs. The remaining 47 out of 17,308 (0.27%) total patients who underwent a primary TJA were diagnosed with an acute PJI (Table 2).

**Table 2:** Demographic data of patients with diagnosed acute periprosthetic joint infections.

Variable	Non-dental clearance	Dental clearance	P-value
	N = 21	N =26	
Body mass index	30.3 (5.60)	31.9 (5.41)	0.317
Age	65.8 (7.98)	69.2 (9.90)	0.190
Sex			0.326
Female	9 (42.9%)	16 (61.5%)	
Male	12 (57.1%)	10 (38.5%)	
Joint			0.473
Hips	12 (57.1%)	11 (42.3%)	
Knees	9 (42.9%)	15 (57.7%)	
Days from surgery	36.9 (21.6)	41.7 (22.0)	0.453
Culture result			0.579
Negative	2 (9.52%)	1 (3.85%)	
Positive	19 (90.5%)	25 (96.2%)	
Procedure type			0.516
Irrigation and debridement with polyethylene exchange	13 (61.9%)	17 (65.4%)	
Resection and spacer	4 (19.0%)	7 (26.9%)	
One-stage revision	4 (19.0%)	2 (7.69%)	

Acute PJI was diagnosed in 26 (0.30%) and 21 (0.24%) patients who did and did not obtain preoperative dental clearance, respectively. No significant statistical difference was seen in the rate of PJIs between the two groups (p = 0.559). The diagnosis was made at a mean of 36.9 days postoperative in the NC group and 41.7 days in the DC group. With the number of patients included in each group, our study had the statistical power to reveal any change in the rate of infection that is greater than 0.081%.

All patients diagnosed with PJIs underwent surgical intervention. Of the 26 patients in the DC group, 17 (65.4%) underwent irrigation and debridement with polyethylene spacer exchange (I&D), two (7.7%) underwent a single-stage revision arthroplasty, and seven (26.9%) underwent a resection arthroplasty with antibiotic spacer implantation. Similarly, the NC group had 13 (61.9%) I&D procedures, four (19.0%) single-stage revisions, and four (19.0%) resection arthroplasties with antibiotic space implantation. No statistical significance was seen between treatment modalities (p = 0.516).

Positive cultures resulted in all but two (4.2%) patients with PJIs. No organisms consistent with oral flora were identified as the causative organism in either group. Antibiotic coverage was prescribed for patients based on microorganism speciation. There was no statistical significance in the PJI causative organism or in the antibiotics administered. Ten cultures were polymicrobial, two (20%) were in the NC group, and eight (80%) were in the DC group. Microorganisms isolated from cultures can be seen in Table 3.

**Table 3:** Organisms identified from periprosthetic joint infection cultures.

Organism	Non-dental clearance	Dental clearance
Methicillin-susceptible *Staphylococcus aureus* (MSSA)	10 (41.7%)	8 (19.5%)
Methicillin-resistant *Staphylococcus aureus* (MRSA)	3 (12.5%)	3 (7.3%)
Staphylococcus epidermidis	3 (12.5%)	1 (2.4%)
Seronegative	2 (8.3%)	0 (0.0%)
Enterococcus faecalis	1 (4.2%)	4 (9.8%)
Streptococcus agalactiae	1 (4.2%)	4 (9.8%)
Proteus mirabilis	1 (4.2%)	3 (7.3%)
Enterobacter cloacae	1 (4.2%)	1 (2.4%)
Staphylococcus warneri	1 (4.2%)	0 (0.0%)
*Streptococcus *(Group G)	1 (4.2%)	0 (0.0%)
Pseudomonas aeruginosa	0 (0.0%)	4 (9.8%)
Candida albicans	0 (0.0%)	2 (4.9%)
Staphylococcus capitis	0 (0.0%)	2 (4.9%)
Staphylococcus lugdunesis	0 (0.0%)	2 (4.9%)
Acinetobacter	0 (0.0%)	1 (2.4%)
Corynebacterium	0 (0.0%)	1 (2.4%)
Globicatella	0 (0.0%)	1 (2.4%)
Klebsiella pneumoniae	0 (0.0%)	1 (2.4%)
Oligella urethralis	0 (0.0%)	1 (2.4%)
Serratia marcescens	0 (0.0%)	1 (2.4%)
Staphylococcus pseudintermedius	0 (0.0%)	1 (2.4%)
Group A *Streptococcus*	0 (0.0%)	0 (0.0%)

The most common organism isolated was methicillin-susceptible *Staphylococcus aureus* (MSSA) for the NC (41.7%) and DC (19.5%) groups. The second most common organisms for the NC group were methicillin-resistant *Staphylococcus aureus* (MRSA; 12.5%) and *Staphylococcus epidermidis* (12.5%). Comparatively, MRSA was the third most common organism for the DC group (7.3%), while the second most common organisms were *Enterococcus faecalis* (9.8%), *Streptococcus agalactiae* (9.8%), and *Pseudomonas aeruginosa* (9.8%). No PJIs were attributable to oral flora, such as *Streptococcus viridans*, *Peptostreptococcus*, *Prevotella*, *Fusobacterium*, *Actinomyces*, *Capnocytophaga*, *Selenomas*, and *Veillonella* [8,21].

Four surgeons during the study period changed their preoperative protocol to no longer include routine dental clearance before elective TJA. The rates of PJIs were compared before and after this change and demonstrated no statistically significant difference in acute PJI rates between NC and DC groups (Table 4).

**Table 4:** Rate of periprosthetic joint infections before and after the dental clearance protocol change.

Variable	Non-dental clearance	Dental clearance	P-value
Surgeon 1	N = 106	N = 1,617	1.000
No	106 (100%)	1,611 (99.6%)	
Yes	0 (0.00%)	6 (0.37%)	
Surgeon 2	N = 870	N = 1,346	0.387
No	867 (99.7%)	1,344 (99.9%)	
Yes	3 (0.34%)	2 (0.15%)	
Surgeon 3	N = 767	N = 31	1.000
No	765 (99.7%)	31 (100%)	
Yes	2 (0.26%)	0 (0.00%)	
Surgeon 4	N = 618	N = 208	1.000
No	616 (99.7%)	207 (99.5%)	
Yes	2 (0.32%)	1 (0.48%)	

## Discussion

PJIs pose a serious burden to patients and the healthcare system. Efforts to minimize the risk of PJI following TJA may include routine preoperative dental clearance to eradicate any active periodontal disease that could potentially cause PJI. However, dental clearance can delay surgery and poses an additional burden to patients. Our study demonstrated that routine dental clearance did not decrease the rate of acute PJIs. and those who did develop a PJI were not caused by oral flora.

Kohler et al. found that 18.5% of patients were required to undergo invasive dental procedures following routine dental screening before elective TJA which represented additional cost and time for patients [11]. Another study by Tokarski et al. found that routine dental clearance resulted in a 12% failure rate (requiring treatment for dental infection), but selective dental clearance based on risk factors (tobacco use, narcotic use, and no dental visit within 12 months) reduced the failure rate to 6% [12]. Lampley et al. demonstrated that patients undergoing elective TJA did not have a significantly greater rate of acute postoperative infections when compared to patients undergoing THA or hemiarthroplasty for hip fractures that did not receive preoperative dental clearance [13]. Additionally, no previous study demonstrated a clinical reduction in infection rate warranting delaying cases, which is consistent with meta-analyses performed by Frey et al. where the authors concluded that universal dental screening before TJA was not recommended [14]. Moreover, Sonn et al. demonstrated no significant differences in complication rates between patients who did and did not receive dental clearance but did find that patients who underwent dental extraction before TJA did have a higher hazard ratio [17].

Our findings concur with previous literature that routine dental clearance and subsequent dental procedures do not appear to reduce the risk of PJI following elective TJA. Patients in the DC and NC groups with confirmed acute PJIs following elective TJA had no significant differences in the rates of PJIs. The causative organism was not found to be oral flora in any of the PJI cases. Surgeons who changed their preoperative elective TJA protocol did not have a significantly different rate of PJIs when comparing patients who received preoperative dental clearance and those who did not.

We recognize that our study has several limitations, most of which are related to its retrospective design. First, our matched cohort was created from a limited number of variables creating the potential for selection bias. Although we created the matched cohort using variables that were felt to be relevant to the study, this does not completely eliminate the potential for selection bias. Second, preoperative dental clearance protocols were not standardized as patients were treated by different providers within different regions of our institution and relied on self-reported preoperative dental screen protocols. As a result, the study design introduces the potential for surgeon recall bias that could lead to inaccurate reporting of their protocols. We mitigated this limitation by thoroughly identifying the surgeon’s true protocols in the timeframe of the study, and after reviewing 10% charts, we found zero inaccuracies. Reviewing 17,308 charts to confirm the surgeon-reported protocols for every case would have required substantial time and energy. After reviewing 1,730 charts (10%) and finding zero discrepancies, it was decided to stop the chart reviews for this purpose due to the substantial time and effort involved. If discrepancies had been found, the chart reviews would have continued. Third, it is possible that patients were unintentionally excluded from the PJI group, as they were identified through billing codes. However, patients who were identified as part of the PJI group underwent an EMR review to confirm acute PJIs, and those who had a suture abscess or unrelated complication were appropriately excluded. Lastly, our study only had the statistical power to detect greater than a 30% change in the infection rate between the groups. Yet, this corresponds to a very low absolute change in the infection rate of 0.081%, making our results clinically meaningful.

It is important to note that a prospective randomized trial that seeks to investigate the potential benefit of preoperative dental clearance is challenging. Because of the low rate of PJI after joint arthroplasty procedures, any prospective study investigating the effect of an intervention on the rate of PJI inherently requires a large number of patients. In addition, dental clearance has significant costs to the patient, surgeon, and practice which would create a large burden for such a study. These factors explain the paucity of prospective studies of preoperative dental clearance. While our study has the limitations inherent to a retrospective study, it has sufficient numbers to suggest that routine preoperative dental clearance does not decrease the rates of acute PJIs following elective TJA and that infections caused by oral flora are not more common when preoperative dental clearance is not obtained.

## Conclusions

PJIs are a serious complication of TJA that place significant psychological and financial burdens on patients and healthcare systems. One measure to reduce the risk of PJIs is preoperative dental screening, for which there is no current consensus recommendation. The purpose of our study is to determine whether there is a difference in the rate of PJIs and microorganism profiles in patients who did and did not obtain preoperative dental clearance. Our retrospective study did not find that dental clearance decreases the rate of infection and also did not show a difference in organism profile between infections after dental clearance versus no dental clearance. Although our study has limitations, with our results, and without strong evidence in the literature to suggest the opposite result, it is reasonable to eliminate dental clearance as a standard practice before TJA.
